# Early induction of oxidative stress in a mouse model of Alzheimer’s disease with heme oxygenase activity

**DOI:** 10.3892/mmr.2014.2252

**Published:** 2014-05-20

**Authors:** SANLI XING, DINGZHU SHEN, CHUAN CHEN, JIAN WANG, ZHIHUA YU

**Affiliations:** Shanghai Geriatric Institute of Chinese Medicine, Shanghai University of Traditional Chinese Medicine, Shanghai 200031, P.R. China

**Keywords:** hippocampus, Alzheimer’s disease, heme oxygenase 1, heme oxygenase 2

## Abstract

Evidence suggests that brain tissues of patients with Alzheimer’s disease (AD) are easily attacked by oxidative stress, and numerous studies indicate that heme oxygenase (HO) is a major cell adaptive responder to stress. However, whether HO-1 and HO-2 play different roles in this process has not yet been studied. In the present study, it was shown in an AD model that HO-1 and HO-2 have different roles in the early stages of AD. Learning and memory ability was tested in APP_swe_/PS1_ΔE9_ (APP/PS1) transgenic and wild-type mice using the Morris water maze. β-amyloid plaques were measured using immunofluorescence staining. Changes in reactive oxygen species (ROS) levels in the hippocampi were measured using a fluorescence technique. The results indicated that the escape latency, amyloid plaque deposition and ROS production increased in the hippocampi of APP/PS1 transgenic mice compared with wild-type mice. Furthermore, using double-immunofluorescence staining and western blot analysis, it was found that the expression of HO-1 and HO-2 increased in the hippocampi of APP/PS1 mice and, notably, HO-2 was also found to be overexpressed in astrocytes. Little difference was observed in the plasma HO-1 concentrations between the two groups, while the plasma HO-2 concentration of the APP/PS1 mice was lower than that of the wild-type mice, shown by ELISA. In conclusion, HO-2 overexpression is an early event and plays a more critical role in the progression of AD.

## Introduction

Alzheimer’s disease (AD) is an age-related neurodegenerative disorder. This disease is pathologically characterized by the deposition of extracellular amyloid-β (Aβ), intracellular neurofibrillary tangles (NFTs) and degenerated neurons. Evidence suggests that brain tissues are continually exposed to oxidative stress during the development of AD (1). Oxidative stress is generally caused by an imbalance between the production of reactive oxygen species (ROS) and the antioxidative defense system, which is considered to be responsible for the decreasing memory (2). Oxidative stress can provoke the cellular adaptive response to injury induced by ROS, but this response often requires the upregulation of endogenous antioxidant enzymes (3). Heme oxygenases (HOs) are considered to be important antioxidant enzymes, which catalyze heme to biliverdin, carbon monoxide and ferrous iron (4). The two main isoforms of HO are HO-1 and HO-2. HO-1 expression is limited to a small number of neurons and glial cells (5), and HO-2 is constitutively expressed in the normal brain (6). It has previously been shown that the expression of HO-1 is increased, while that of HO-2 is reduced, in the brains of patients with AD (7). In addition, it has been shown that the sustained upregulation of HO-1 may contribute to the pathological iron deposition, oxidative damage and mitochondrial dysfunction in AD (8).

Previous studies have shown that is an association between HO expression and AD (9,10), but whether HO-1 and HO-2 have different roles in the early stages of AD has not yet been studied. In the present study, using the APP_swe_/PS1_ΔE9_ (APP/PS1) transgenic mouse model, HO-1 and HO-2 expression was monitored with immunofluorescence and western blotting methods. Furthermore, the differential expression patterns of HO-1 and HO-2 in neuronal and glial cells were observed using immunofluorescence methods, and the plasma concentrations of HO-1 and HO-2 were determined by ELISA.

## Materials and methods

### Experimental animals

Six-month-old male APP/PS1 transgenic and wild-type mice were used in this study, with 12 mice per group. The APP/PS1 mouse strain is a double-transgenic hemizygote that expresses a chimeric mouse/human amyloid precursor protein and mutant human presenilin-1. APP/PS1 transgenic mice were purchased from the Model Animal Research Center of Nanjing University (Nanjing, China). Wild-type mice were purchased from the Experimental Animal Center of Shanghai Academy (Shanghai, China). All procedures were performed in accordance with the Guide for the Care and Use of Medical Laboratory Animals (Ministry of Health, China, 1998) and the guidelines of the Shanghai University of Traditional Chinese Medicine (Shanghai, China) Laboratory Animal Care and Use Committee.

### Morris water maze test

Since the Morris water maze is a test evaluating spatial learning (11), the test was selected in the present study to assess hippocampus-dependent spatial learning and memory. The water maze was divided into four equal quadrants and a hidden platform was submerged 1 cm beneath the water surface. The water temperature was kept between 20 and 23°C. Each mouse was tested in four trials per day with an inter-trial interval of 30–40 min, which continued for 5 days. In each trial, the mouse was released facing the wall of the pool from one of four starting points (north, east, south or west). The mouse was allowed to search for the platform for ≤70 sec, allowing it to rest 30 sec on the platform. The time the mice spent finding the platform was recorded as the escape latency. The experiments were recorded with a camera connected to a video recorder and a computerized tracking system.

### Measurement of ROS levels

Intracellular ROS levels of the hippocampus were measured as previously described (12). Briefly, hippocampi were homogenized in 0.01 M phosphate-buffered saline (PBS) (pH 7.2–7.4). The homogenized cells (0.4 mg/ml) were loaded with the cell permeant probe 2,7-dichlorofluorescein diacetate (DCFH-DA, 20 μM) for 60 min at 37°C in the dark, then the fluorescence was measured through a spectrofluorometer (Synergy HT, BioTek Instruments, Winooski, VT, USA) using 485 nm as the excitation and 525 nm as the emission wavelength. The normalized data were expressed as a value of 100%.

### Immunofluorescence analysis

Mice were anesthetized with 5% chloral hydrate and perfused through the heart with saline solution followed by 4% paraformaldehyde. The brains were then removed, post-fixed in 4% paraformaldehyde for 24 h and immersed in 30% sucrose until they sank. Thereafter, coronal sections (30 μm) were cut using a freezing microtome (Microm™ HM 525 Cryostat, Thermo Scientific, Walldorf, Germany). Sections were permeabilized with 0.2% Triton X-100, then blocked in 5% bovine serum albumin for 30 min. The sections were subsequently incubated with primary antibodies, including polyclonal anti-Aβ_1-42_ (1:200; Abcam, Cambridge, MA, USA), monoclonal anti-HO-1 (1:200; Abcam), monoclonal anti-HO-2 (1:100; Santa Cruz Biotechnology, Inc., Dallas, TX, USA), monoclonal anti-Neuronal Nuclei (1:500; Millipore, Billerica, MA, USA) and monoclonal anti-glial fibrillary acidic protein (GFAP; 1:500; Abcam), overnight at 4°C. Having been washed with 0.01 M PBS three times, the sections were then incubated with fluorescein isothiocyanate (1:200; Santa Cruz Biotechnology, Inc.) or Cy3^®^-conjugated secondary antibodies (1:200; Abcam) at 37°C for 1 h. Fluorescent signals were detected by fluorescence microscopy.

### Western blotting

Total protein concentration was determined using a Micro BCA™ Protein Assay kit (Pierce Biotechnology, Inc., Rockford, IL, USA). Protein samples were resolved in SDS sample buffer. The protein samples of ~30 mg were run on a 10% SDS-PAGE gel and the protein in the gel was transferred onto polyvinylidene fluoride membrane. The membranes were incubated with anti-HO-1 (1:2,000; Abcam), anti-HO-2 (1:1,000; Abcam) and anti-GAPDH (1:5,000; Abcam) primary antibodies at 4°C overnight. The membranes were washed with Tris-buffered saline Tween-20 buffer three times every 10 min. The membranes were incubated with IRDye 800CW (Li-Cor, Inc., Lincoln, NE, USA) secondary antibodies for 1 h at room temperature and the blots were visualized using an Odyssey^®^ scanner (Li-Cor, Inc.).

### ELISA

Briefly, 100 μl diluted plasma was loaded onto 96-well plates. Levels of HO-1 and HO-2 were determined using an ELISA kit (Shanghai Westtang Bio-tech, Shanghai, China) according to the manufacturer’s instructions.

### Statistical analysis

Statistical analysis was performed using GraphPad Prism version 5 software (GraphPad Software, Inc., La Jolla, CA, USA). Measurement data are expressed as the mean ± standard error of the mean. Differences were assessed using the Student’s t-test for comparisons, A value of P<0.05 was considered to indicate a statistically significant difference.

## Results

### Memory impairment in APP/PS1 transgenic mice determined through Morris water maze analysis

**Figure 1:**
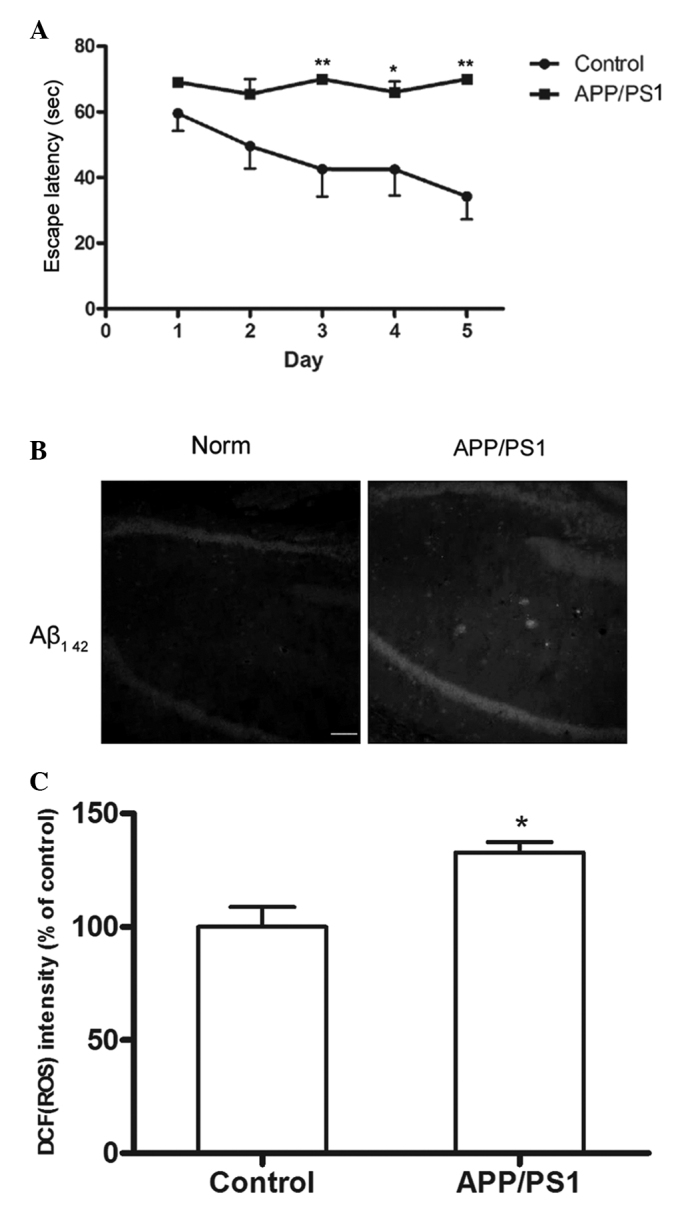
Spatial learning and memory, amyloid protein deposition and ROS generation in APP/PS1 mice. (A) Latency to escape onto a hidden platform in the Morris water maze (n=12). ^*^P<0.05, ^**^P<0.01 versus control. (B) Immunofluorescent micrographs of amyloid plaques (scale bar, 50 μm; original magnification, ×100). Amyloid plaques can be observed in the hippocampi of 6-month-old APP/PS1 mice; these are rarely found in wild-type mice at the same age. (C) ROS levels (DCF fluorescence) in the hippocampus of APP/PS1 transgenic mice (n=4). ^*^P<0.05 versus control. ROS, reactive oxygen species; DCF, 2,7-dichlorofluorescein; Aβ, amyloid-β.

The Morris water maze was employed to detect the memory ability of the 6-month-old APP/PS1 transgenic mice. It was found that the mean latency of the APP/PS1 group became significantly longer than that of the control group between the third and the fifth day (P<0.05; Fig. 1A). The results showed that the spatial learning and memory of the APP/PS1 transgenic mice was impaired.

### Increased Aβ plaques and ROS levels in the hippocampi of APP/PS1 transgenic mice

Small, diffuse Aβ deposits were observed in the hippocampus, but not in the cortex, of APP/PS1 transgenic mice; this was due to the fact that the transgenic mice used were only 6 months old. Less Aβ staining was observed in the hippocampus or cortex of wild-type mice (Fig. 1B). In the present study, the generated ROS were assessed with the membrane-permeable fluorescent probe DCFH-DA. The ROS levels in the hippocampi of the APP/PS1 transgenic mice were found to be significantly higher than those of the wild-type mice (P<0.05; Fig. 1C).

### Increased HO-1 expression in APP/PS1 transgenic mice

**Figure 2:**
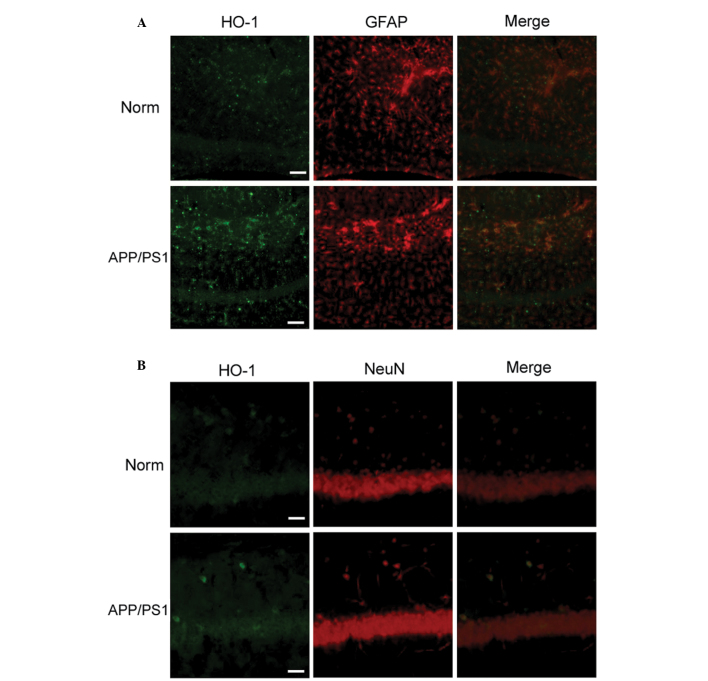
HO-1 expression in 6-month-old APP/PS1 mice (scale bar, 20 μm; original magnification, ×200). (A) Representative photomicrograph of HO-1 immunoreactivity in the hippocampi of APP/PS1 transgenic mice; GFAP-positive astrocytes (red) exhibited HO-1 immunoreactivity (green). (B) Double immunolabeling was performed with antibodies against HO-1 (green) and NeuN (red). Overlapping signals are visualized in yellow. HO-1, heme oxygenase 1; GFAP, glial fibrillary acidic protein; NeuN, Neuronal Nuclei; Norm, wild type.

HO-1 is restricted in expression to small groups of dispersed neurons and glial cells (6). Using immunofluorescence labeling, it was observed that astrocytes exhibited increased HO-1 immunoreactivity in the hippocampi of APP/PS1 transgenic mice compared with that of normal mice. In contrast to the overexpression of HO-1 in GFAP-positive astrocytes (Fig. 2A), it was observed that neurons exhibited faint HO-1 immunoreactivity in the hippocampi of APP/PS1 transgenic mice, but that the immunoreactivity was still higher than that in the control group (Fig. 2B). The result indicated that induced overexpression of HO-1 in astrocytes is an early event in the pathogenesis of AD.

**Figure 3:**
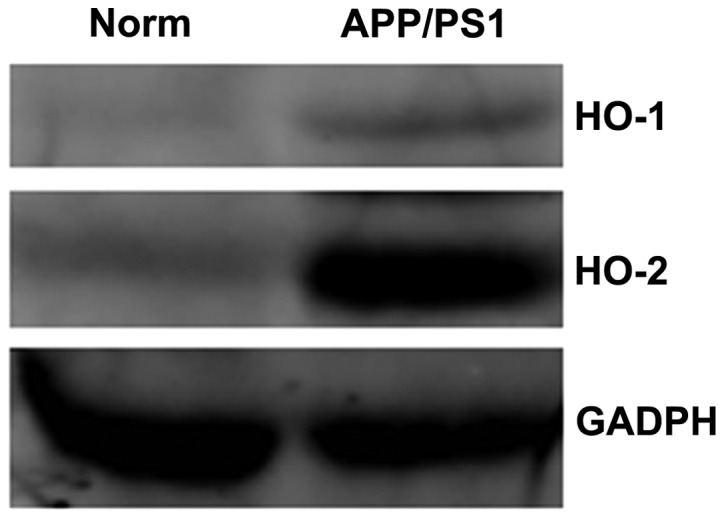
Detection of HO-1 and HO-2 expression in the hippocampi of APP/PS1 transgenic mice (n=3). The hippocampus proteins were detected by western blotting methods with GAPDH as a reference protein. HO, heme oxygenase; Norm, wild type.

For further confirmation of HO-1 overexpression in the hippocampi of APP/PS1 transgenic mice, protein extracts were prepared and analyzed by western blotting. Higher expression levels of HO-1 were observed in the transgenic mice than in the wild-type mice (Fig. 3).

### Increased HO-2 expression in APP/PS1 transgenic mice

**Figure 4:**
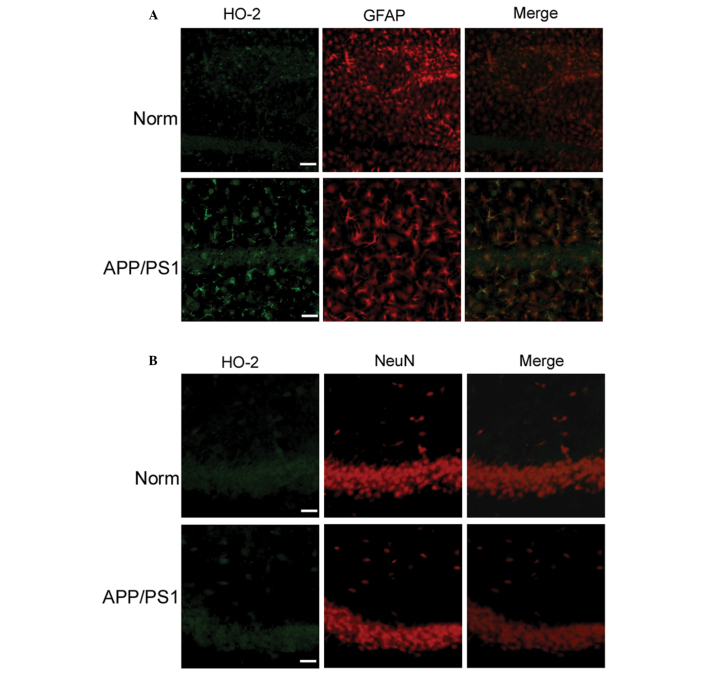
HO-2 expression in 6-month-old APP/PS1 mice (scale bar, 20 μm; original magnification, ×200). (A) Sections were doubled labeled with HO-2 (green) and GFAP (red). (B) Immunofluorescent staining of HO-2 (green) and NeuN (red) in the hippocampi of mice. (scale bar, 20 μm). HO-2, heme oxygenase-2; GFAP, glial fibrillary acidic protein; NeuN, Neuronal Nuclei; Norm, wild type.

As an antioxidant enzyme, HO is markedly induced under conditions of oxidative stress, and several reports have shown that HO-2 accounts for ~80% of the total rodent brain HO activity (13,14). Therefore, the changes in HO-2 in the early stages of AD were investigated in the present study. Using double-label fluorescence microscopy, it was observed that the hippocampi of APP/PS1 transgenic mice exhibited more numerous immunoreactive GFAP cells coexpressing HO-2 protein than the control mice (Fig. 4A); however, HO-2 immunoreactivity was hardly detectable in neurons of transgenic and wild-type mice (Fig. 4B). In order to confirm the overexpression of HO-2 in the hippocampi of APP/PS1 transgenic mice, protein extracts were prepared and analyzed by western blotting. HO-2 expression was shown to be higher in the APP/PS1 transgenic mice than that in the wild-type mice (Fig. 3). In addition, more GFAP-positive astrocytes showing HO-2 immunoreactivity were observed than those exhibiting HO-1 immunoreactivity. These results indicated that HO-2 has a more important role in the early stages of AD.

### HO-1 and HO-2 concentration in plasma

**Figure 5:**
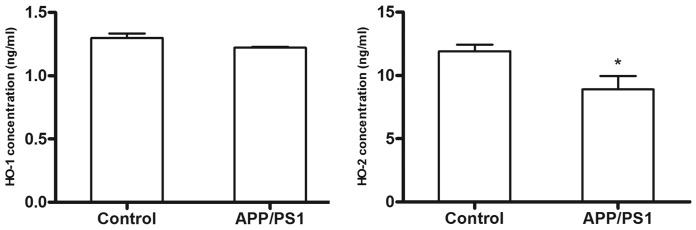
HO-1 and HO-2 concentration in plasma. Plasma HO-1 and HO-2 levels were measured by ELISA (n=6). ^*^P<0.05 versus control. HO, heme oxygenase.

In contrast to the overexpression of HO-1 protein in the brains of the 6-month-old APP/PS1 transgenic mice, the plasma HO-1 concentration in the transgenic mice showed little difference from that in the control mice, as determined by ELISA (P>0.05). In addition, unlike the overexpression pattern of HO-2 in the brains of the transgenic mice, the plasma HO-2 concentration in the transgenic mice was lower than that of the control group (P<0.05). Since the results of plasma HO-2 concentration were not consistent with those for the AD brain (Fig. 5), it was hypothesized that the HO-2 suppressor activity was more robust than the HO-2 activity in the plasma of AD mice models.

## Discussion

AD is recognized as a clinical pathological disease with multiple causes, including genetic, environmental and lifestyle factors (15); oxidative stress may be one of the underlying factors contributing to the progression of AD. Oxidative stress is considered to be the equilibrium state where the production of ROS exceeds the capability of the antioxidant systems (16). The brain is an organ rich in cholesterol and polyunsaturated fatty acids and is vulnerable to oxidative stress (17–20). In a previous study, products of oxidative stress were found in senile plaques or NFTs (21). As oxidative stress can be induced by deposited amyloid peptide and accumulated tau (22,23), it was speculated in the present study that endogenous antioxidant systems play important roles in the pathogenesis of AD. The HO system is one of the major endogenous antioxidant systems involved in AD (24,25).

In a previous study, Huang *et al* (26) found that injection of Aβ into the brains of adult Sprague Dawley rats could markedly reduce the expression of HO-1; however, the present study indicated that HO-1 expression was increased in the hippocampi of 6-month-old APP/PS1 transgenic mice, which was consistent with a study by Schipper *et al* (10). At a later stage, it is likely that HO-1 overexpression began facilitating the deposits of NFTs and amyloid protein due to toxic product of heme metabolism (27,28).

The main roles of oxidative stress in the pathogenesis of AD have been largely investigated, and numerous studies have reported that plasma levels of oxidative products are increased in patients with AD compared with controls (29–31). In support of the hypothesis that plasma antioxidant capacity is impaired in AD, the present study investigated the HO concentrations in the plasma of AD disease models. A significant reduction in HO-2 plasma concentration was observed in 6-month-old transgenic mice compared with age-matched controls, but plasma HO-1 concentration remained unchanged. These results indicated that HO-2 may play a more important role than HO-1 in the pathological process of AD.

The present results partially supported the study by Barone *et al* (7) on subjects with AD (particularly HO-1 data); however, the present HO-2 data were not in agreement with those from patients with AD. Plasma HO-1 and HO-2 concentrations may provide a novel insight that the induction of antioxidant capacity, particularly HO-2, is an early event in the progression of AD.

## Abbreviations

AD: Alzheimer’s disease
Aβ: amyloid-β
NFT: neurofibrillary tangles
ROS: reactive oxygen species
HO: heme oxygenase
CO: carbon monoxide
